# Generation of human TMEM16F-specific affibodies using purified TMEM16F

**DOI:** 10.3389/fmolb.2023.1319251

**Published:** 2024-01-11

**Authors:** Eunyoung Kim, Jinho Bang, Ji Hye Sung, Jonghwan Lee, Dae Hwan Shin, Sunghyun Kim, Byoung-Cheol Lee

**Affiliations:** ^1^ Korea Brain Research Institute, Neurovascular Unit Research Group, Daegu, Republic of Korea; ^2^ Korea Institute of Ceramic Engineering and Technology, Bio-Healthcare Materials Center, Cheongju, Republic of Korea; ^3^ College of Pharmacy, Chungbuk National University, Cheongju, Republic of Korea

**Keywords:** phospholipids, scramblase, TMEM16, affibody, biopanning

## Abstract

**Introduction:** TMEM16 family proteins are involved in a variety of functions, including ion transport, phospholipid scrambling, and the regulation of membrane proteins. Among them, TMEM16F has dual functions as a phospholipid scramblase and a nonselective ion channel. TMEM16F is widely expressed and functions in platelet activation during blood clotting, bone formation, and T cell activation. Despite the functional importance of TMEM16F, the modulators of TMEM16F function have not been sufficiently studied.

**Method:** In this study, we generated TMEM16F-specific affibodies by performing phage display with brain-specific TMEM16F (hTMEM16F) variant 1 purified from GnTi^−^ cells expressing this variant in the presence of digitonin as a detergent. Purified human TMEM16F protein, which was proficient in transporting phospholipids in a Ca^2+^-dependent manner in proteoliposomes, was coated onto plates and then the phage library was added to fish out TMEM16F-binding affibodies. For the validation of interaction between affibodies and TMEM16F proteins, ELISA, bio-layer interferometry, and size exclusion chromatography were conducted.

**Results and Discussion:** As a result, the full sequences of 38 candidates were acquired from 98 binding candidates. Then, we selected 10 candidates and purified seven of them from *E. coli* expressing these candidates. Using various assays, we confirmed that two affibodies bound to human TMEM16F with high affinity. These affibodies can be useful for therapeutical and diagnostic applications of TMEM16F-related cancer and neurodegenerative diseases. Future studies will be required to investigate the effects of these affibodies on TMEM16F function.

## 1 Introduction

Lipid composition is asymmetrically distributed in the outer and inner leaflets of the plasma membrane (Pomorski and Menon, 2006). For instance, phosphatidylserine (PS) and phosphatidylethanolamine (PE) are mainly located in the inner leaflet. This asymmetry is generated and maintained by two ATP-driven pumps called flippase and floppase (Pomorski and Menon, 2006; Bevers and Williamson, 2010). Lipid scrambling is a rapid process that disrupts the asymmetric distribution of lipids and occurs bi-directionally between leaflets though the action of lipid scramblases (Pomorski and Menon, 2006). Changes in the distribution of lipids by lipid scramblases can produce critical signals (Bevers and Williamson, 2010), the best known of which is the “eat-me” signal on apoptotic cells (Balasubramanian and Schroit, 2003). Cells fated for cell death expose PS on their outer leaflets, and then PS is recognized by phagocytic cells such as macrophages or microglia cells, which remove the dead cells by phagocytosis. During the last few decades, numerous studies have attempted to understand the mechanism of Ca^2+^-activated lipid scramblase, a member of the TMEM16 family of proteins (Ehlen et al., 2013).

TMEM16 proteins comprise 10 family members and were initially reported as Ca^2+^-activated Cl-channels (CACC) (Caputo et al., 2008; Schroeder et al., 2008; Yang et al., 2008). TMEM16A and TMEM16B possess only CACC activity, while other family members, such as TMEM16C, TMEM16D, TMEM16E, TMEM16F, TMEM16G, TMEM16J, and TMEM16K, have lipid transport activity (Suzuki et al., 2013; Yu et al., 2015; Gyobu et al., 2016; Bushell et al., 2019). A recent study reported that TMEM16H produces a Ca^2+^-activated anion current (Schreiber et al., 2020). TMEM16F is one of the most studied proteins among TMEM16 family members. Patients with mutations and mice with knockout of the TMEM16F gene show malfunction in blood coagulation (Suzuki et al., 2010; Castoldi et al., 2011; Yang et al., 2012; Ehlen et al., 2013; Boisseau et al., 2018). TMEM16F-mediated PS exposure is required for the platelets to aggregate (Yang et al., 2012). TMEM16F also plays important roles in membrane fusion (Zhang et al., 2020; Whitlock and Chernomordik, 2021), membrane repair (Wu et al., 2020), viral infection (Zaitseva et al., 2017; Sim et al., 2022), and phagocytosis by macrophages (Ousingsawat et al., 2015) and microglial cells (Batti et al., 2016; Zhao and Gao, 2019). Furthermore, scramblase-induced changes in lipid composition produce signaling events in cancer cells. In tumors, PS exposure triggers immunosuppressive effects on dendritic cells and natural killer cells (Birge et al., 2016). Interestingly, solid tumor and tumorigenic cell lines exhibit high PS exposure on the outer leaflet (De et al., 2018).

Despite the physiological importance of TMEM16F proteins, little is known about the modulators of TMEM16F proteins. Polyphenol compounds previously reported as TMEM16F inhibitors turned out to simply quench the signal of the fluorescent material used in the assays and thus are no longer considered true modulators of TMEM16F function (Le et al., 2020). In a recent report, drugs that blocked the formation of syncytium, which is specifically found in the lungs of patients who die of COVID-19, were screened for using FDA/EMA approved libraries (Braga et al., 2021). One of the most effective drugs identified was niclosamide, an anthelmintic that is effective against cestode infection in humans. Niclosamide was found primarily to block the fusion of spike-forming cells by inhibiting the function of TMEM16F protein (Braga et al., 2021). Subsequent cryo-EM studies revealed that niclosamide and 1PBC binds to the hydrophobic groove of TMEM16F and inhibits both TMEM16F and TMEM16A (Feng et al., 2023). Recently, another study showed that A6-001, a potent TMEM16F inhibitor, inhibits SARS-Cov-2 Spike pseudotyped virus (SARS2-PsV) infection and SARS-Cov-2 viral replication in various cells (Sim et al., 2022). However, it is still controversial whether the compounds directly inhibit the function of the TMEM16F protein or not. For instance, the inhibition of TMEM16F by EGCG, Tannic Acid, Niclosamide, Clofazimine and Ani9 was not observed in *in vitro* assay using proteoliposomes (Zanni et al., 2022). These results suggest that these small molecules do not act directly on the TMEM16F proteins.

Many types of targeting molecules are used in a variety of applications, such as molecular probes for diagnostic imaging, modulators of protein function in targeted therapy, and conjugates for protein purification (Gronwall and Stahl, 2009). These molecules come in diverse forms, such as antibodies, peptides, affibodies, aptamers, and small compounds. Among these, affibodies are rather small molecules of 58 amino acids that contain three helix bundles from the Z-domain of protein A (Nord et al., 1997). Affibodies are extremely stable at high temperatures, such as 90°C, and under acidic and basic conditions (pH 2.5 to pH 11) (Stahl et al., 2017). Affibodies have a much lower molecular weight (6 kDa) than monoclonal antibodies (150 kDa), which makes them suitable for diagnostic imaging. Additionally, molecules smaller than 10 kDa can reach target regions more easily because their low molecular mass allows them to leak rapidly from blood vessels and penetrate tissues (Schmidt and Wittrup, 2009). Affibodies identified through *in vitro* selection methods, such as phage display, can specifically target a wide range of proteins with affinities spanning the micromolar (μM) to picomolar (pM) range (Mouratou et al., 2015). With applications across various fields including diagnostic imaging, targeted therapy, and protein purification, affibodies stand as versatile and promising tools in research and biotechnology (Feldwisch and Tolmachev, 2012).

In this study, we developed novel affibodies that target human TMEM16F proteins using purified hTMEM16F protein to fish out candidate affibodies from bacteriophage libraries. The interaction of each affibody with hTMEM16F protein was validated in various independent experiments.

## 2 Results

### 2.1 Protein purification and functional validation of TMEM16F

**FIGURE 1:**
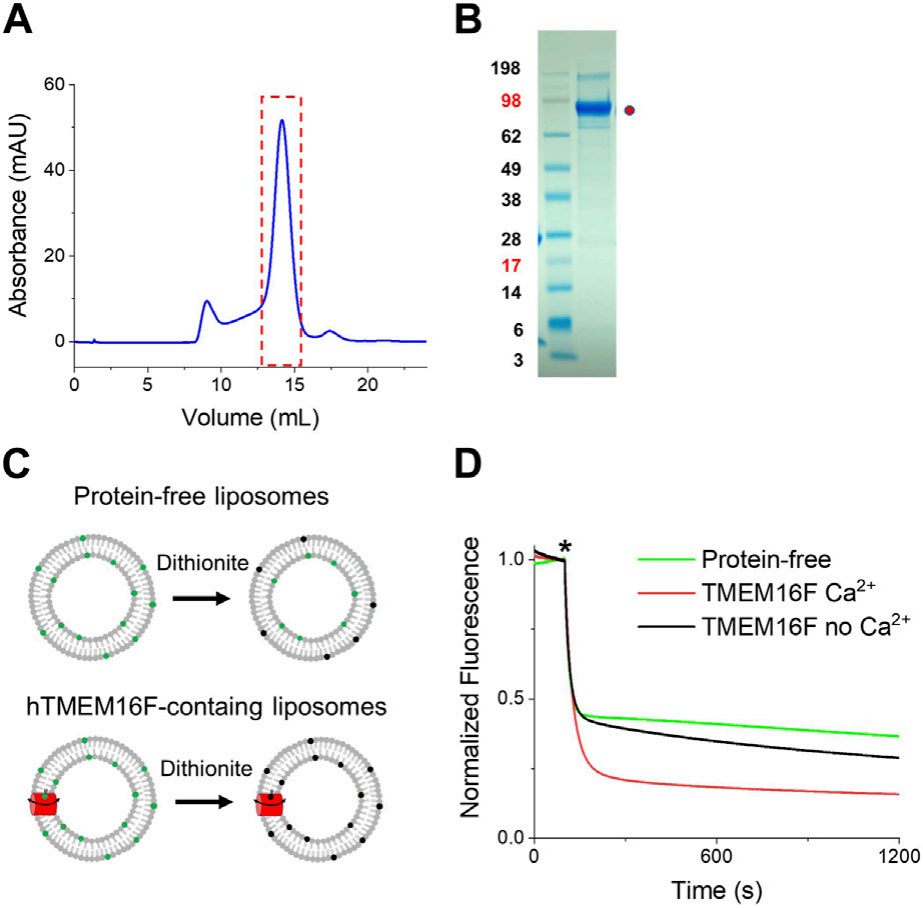
Purification of human TMEM16F and functional validation of scrambling activity **(A)** FPLC profile of purified TMEM16F proteins. After purifying human TMEM16F using digitonin, SEC was performed on a Superose 6 column. **(B)** SDS-PAGE and Coomassie blue staining of hTMEM16F after FPLC. **(C)** Schematic diagram of the scrambling assays for protein-free (*upper*) and proteoliposomes (*lower*). **(D)** Representative traces of dithionite-induced fluorescence decay for hTMEM16F in the presence (red) and absence (black) of Ca^2+^. Protein-free traces are shown in green.

To screen for new affibodies that target TMEM16 scramblases, human TMEM16F (hTMEM16F) variant 1 was purified using affinity chromatography. Briefly, a construct expressing TMEM16F linked to streptavidin binding peptide (SBP) was transfected into 293 cell (GnTi^−^), and TMEM16F proteins were extracted in the presence of digitonin as a detergent. After elution from the streptavidin-conjugated resin, proteins were separated by size exclusion chromatography (SEC). A monodisperse peak of human TMEM16F protein was eluted around 14 mL, and was collected and subjected to SDS-PAGE. Most of the protein migrated near 98 kDa (Figures 1A, B). The functional activity of hTMEM16F was validated by conducting scrambling assays. Trace amounts of fluorescent-labeled phospholipids were added to a lipid mixture (3POPC, 1-Palmitoyl-2-oleoyl-sn-glycero-3-phosphocholine:1POPG, 1-Palmitoyl-2-oleoyl-sn-glycero-3-[phospho-rac-(1-glycerol)]) to make liposomes, and then dithionite-induced fluorescence decay was monitored (Figure 1C). In protein-free liposomes, fluorescent signal decay was ∼50% because membrane-impermeable dithionite can only bleach outer leaflet fluorophores (Figures 1C, D). In good agreement with previous results, fluorescent signal decay from proteoliposomes containing hTMEM16F was much higher than that from protein-free liposomes and the decay was Ca^2+^ dependent (Figure 1D). In the presence of Ca^2+^, we observed a faster and stronger signal decrease in fluorescence. These results suggest that purified hTMEM16F is functionally active.

### 2.2 Screening of hTMEM16F-specific affibodies

**FIGURE 2:**
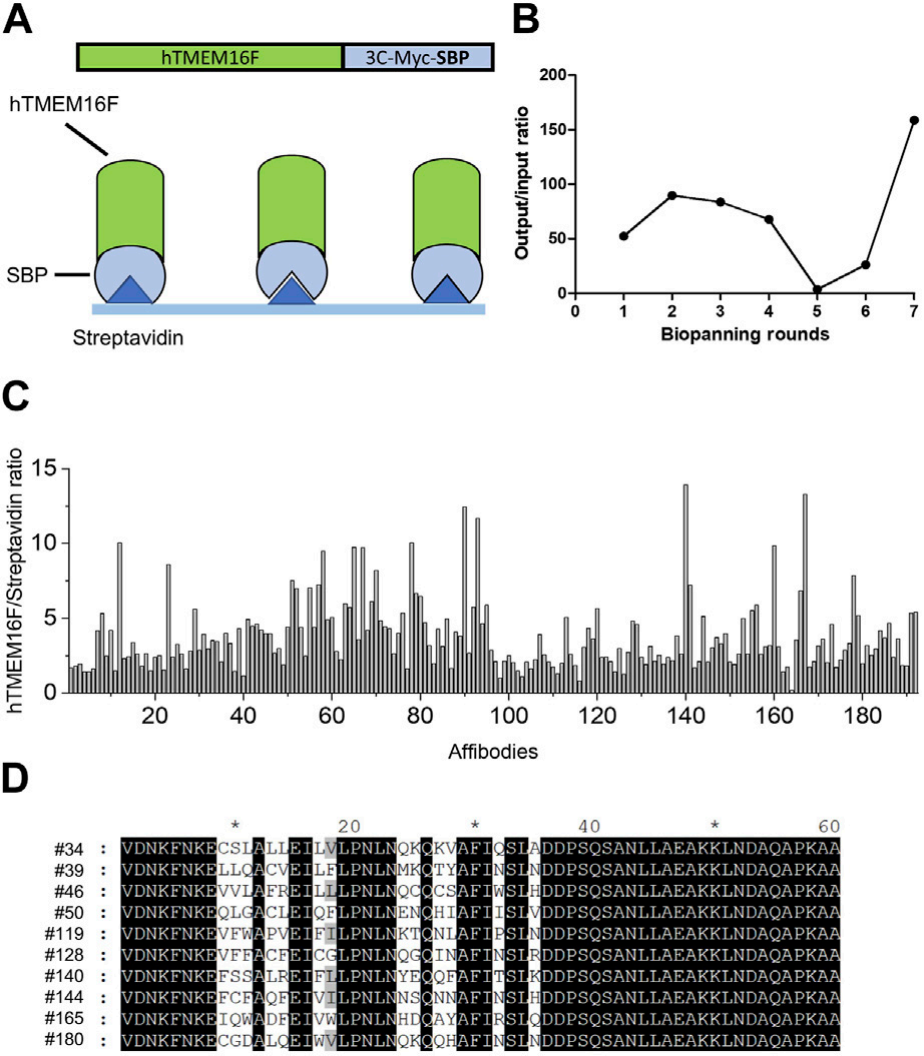
Screening for TMEM16F-specific affibodies using phage display **(A)** Schematic diagram of biopanning for TMEM16F-specific affibodies using streptavidin-coated plates. **(B)** Measurements of output/input phage ratios in all rounds of biopanning. **(C)** hTMEM16F/streptavidin ratios of the 192 candidate affibodies in ELISA from the seventh round of biopanning. **(D)** The amino acid sequences of the selected 10 affibodies.

To screen for affibodies specific for TMEM16F, purified human TMEM16F protein was used for biopanning (Figure 2A). The combinatorial affibody library was composed of 3 × 10^8^ independent affibody clones fused to the N-terminus of the pIII protein of M13 bacteriophage. Seven rounds of biopanning were performed to isolate highly specific affibodies to hTMEM16F. To increase the selection stringency, the number of washes was increased for each round. Significant enrichment of hTMEM16F-binding peptides (160-fold increase) was obtained after seven rounds of biopanning (Figure 2B). Affibody phages (192) from the last round of panning were randomly selected to examine their binding to hTMEM16F using ELISA (Figure 2C). Streptavidin was used as a negative control because it was used to attach TMEM16F protein to the plate via its SBP tag. The 192 individual affibody phages were then incubated on hTMEM16F- and streptavidin-coated plates, and the streptavidin-only-coated plate was used as a background control. After washing with PBS containing 0.05% Tween 20, 98 candidate phages were selected with a hTMEM16F signal to background ratio of more than 2.

### 2.3 Selection of candidate affibodies and their expression

**FIGURE 3:**
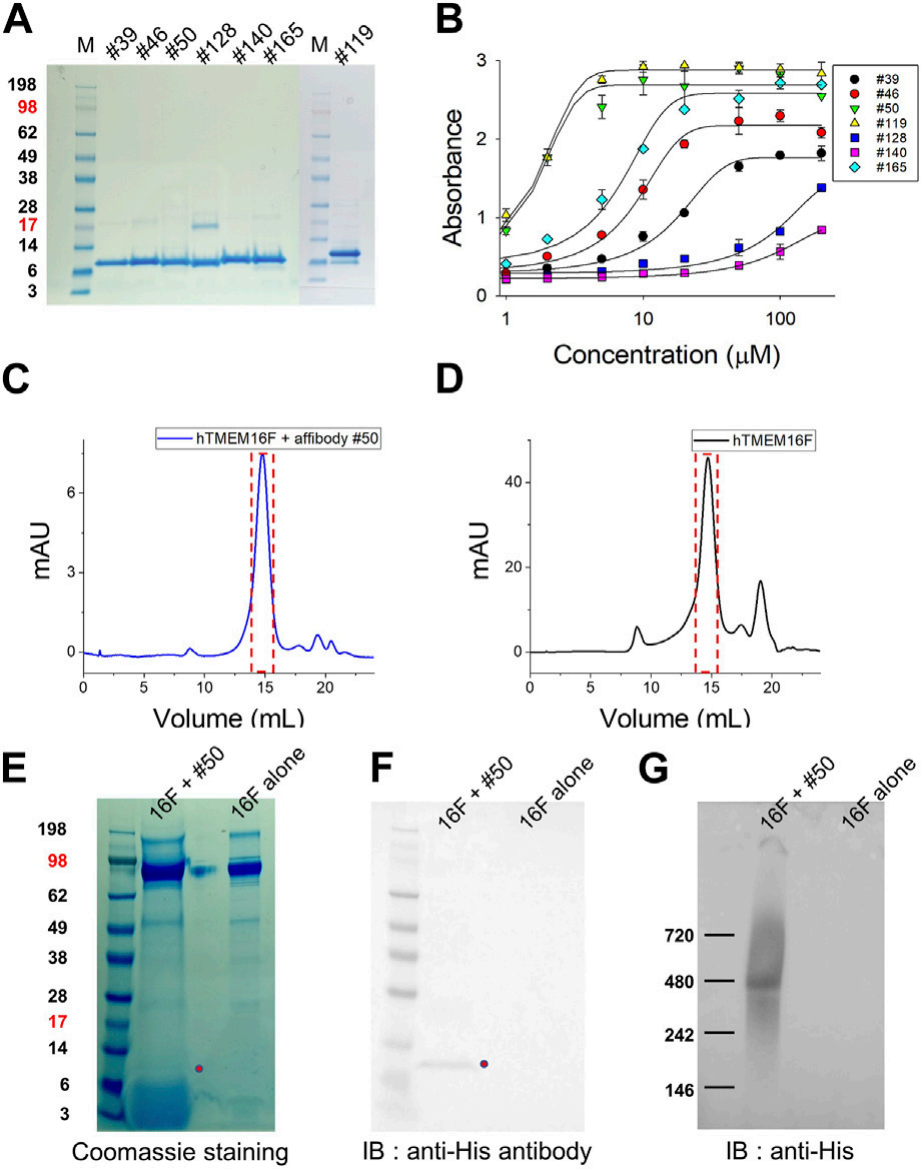
Expression of candidate affibodies and their binding to hTMEM16F proteins. **(A)** The seven selected affibodies were purified and their purity was confirmed by SDS-PAGE and Coomassie blue staining. A total of 10 mg of each affibody was loaded into each lane. **(B)** Verification of affibody binding to hTMEM16F by ELISA, in which each well was coated with 5 mg of purified TMEM16F and then interacted with 1–200 μM of the his-tagged affibody. Superose 6 FPLC elution profile of TMEM16F and #50 affibody together **(C)** and TMEM16F alone **(D)**. The Affibody and TMEM16F were mixed at 4°C for 3 h, and then separated by Superose 6 FPLC. After FPLC, the presence of TMEM16F and the affibody in the same fraction was confirmed by Coomassie blue staining of SDS-PAGE gels **(E)**, Western blotting **(F)** and Blue Native PAGE followed by Western blotting **(G)**.

After verifying the sequences of the 98 candidates, 10 affibodies were selected for further investigation (Figure 2D). The selected affibody genes were synthesized and cloned into a bacterial expression vector with an N-terminal His-tag sequence for purification. Before large-scale culture, the optimal expression conditions for each affibody were defined (Supplementary Figure S1). Affibody expression was induced by IPTG at 37°C and 25°C for 3 h. In small scale cultures, six candidates showed high protein expression, while one candidate showed very low protein expression. All affibodies were more highly expressed at 37°C than at 25°C (Supplementary Figure S1). The remaining three affibodies (#34, #144, and #180) did not show any expression in the presence of IPTG at either temperature (Supplementary Figure S1), even when they were expressed in 1 L cultures. Finally, seven affibodies from 1 L cultures were purified using cobalt column chromatography. Before performing ELISA and Bio-Layer Interferometry (BLI) experiments, the purified proteins were concentrated using a centrifugal filter unit and residual imidazole was removed by dialysis (Figure 3A).

### 2.4 Interaction of candidate affibodies with hTMEM16F protein

Before measuring the binding affinity of each candidate affibody, the interactions between hTMEM16F and the candidate affibodies were validated by ELISA (Figure 3B). After coating a 96-well plate with purified hTMEM16F protein, various concentrations of the seven His-tagged candidate affibodies were added to the wells. The results showed that among the seven affibodies, #50 and #119 exhibited high binding affinity for hTMEM16 protein with an EC_50_ value of 1.6 μM #128 (EC_50_, 83.5 μM) and #140 (EC_50_, 73.3 μM) showed low binding affinity, and their binding did not reach a plateau even when they were added at concentrations up to 200 μM. The remaining three affibodies, #39, #46, and #165, exhibited modest binding affinity for TMEM16F with EC_50_ values of 15.3, 8.0, and 6.1 μM, respectively.

Next, we tested if each of the validated affibodies could bind to native TMEM16F by performing SEC of a mixture of TMEM16F and #50 affibody or TMEM16F alone. After incubating 40 μg of purified TMEM16F (0.4 mg/mL) protein with 40 μg of #50 affibody (0.5 mg/mL) for 2 h at 4°C, the mixture was separated by Superose 6 fast protein liquid chromatography (FPLC) (Figures 3C, D). SDS-PAGE and Western blot analyses of fractions eluted around 14.8 mL revealed the presence of both the #50 affibody and the TMEM16F dimer with a molecular weight above 200 kDa (Figures 3E, F). In Blue Native PAGE followed by Western blot, we also confirmed that #50 affibody could co-migrate with TMEM16F near 480 kDa as reported in other studies (Ishihara et al., 2016; Watanabe et al., 2018) (Figure 3G; Supplementary Figure S2). These data strongly suggest that this affibody binds to native TMEM16F protein.

**FIGURE 4:**
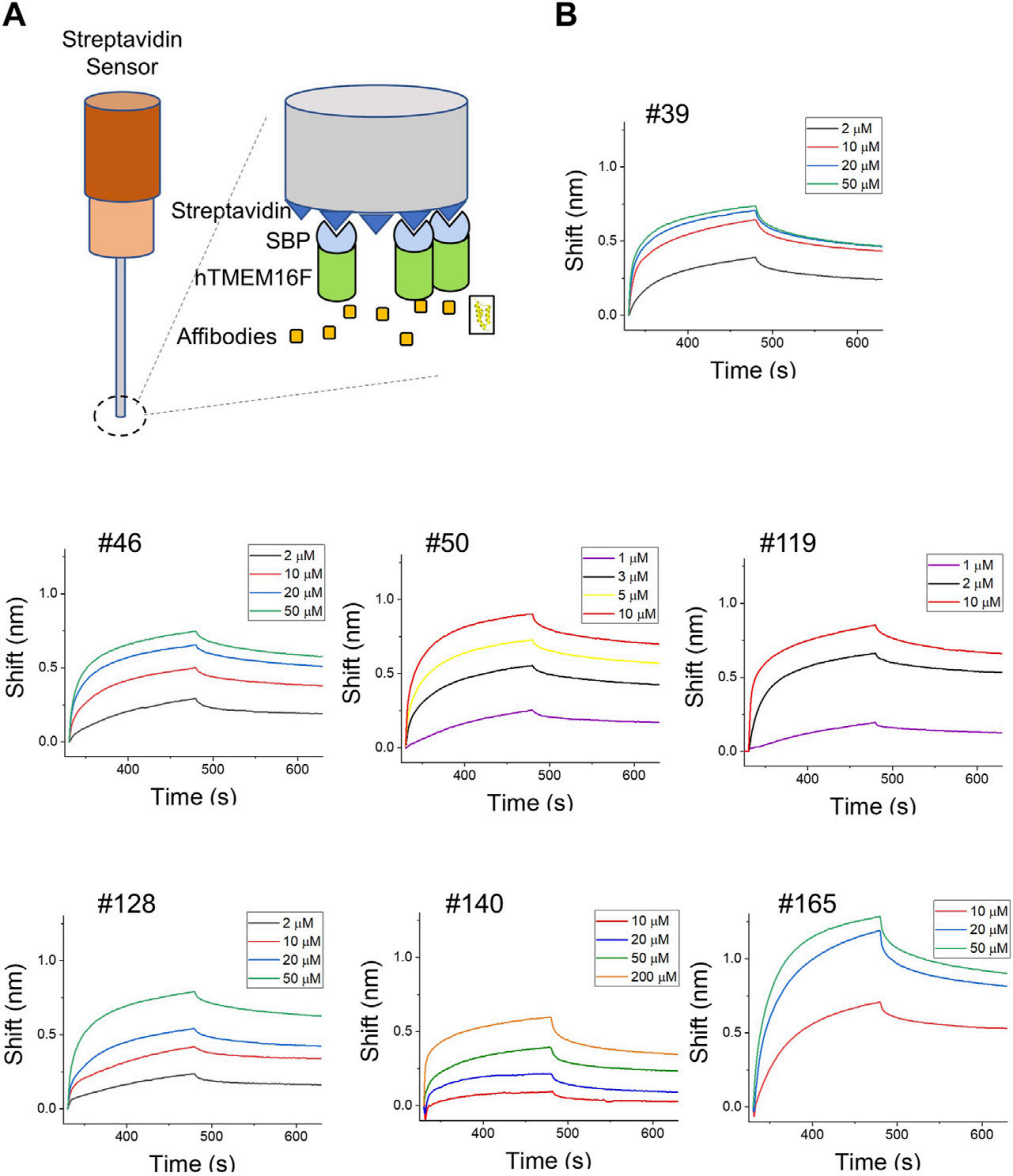
Direct measurement of binding affinities using BLI assays. **(A)** Schematic diagram of the BLI assay using SBP-tagged hTMEM16F. **(B)** BLI instrument measurements of the binding affinities of candidate affibodies. TMEM16F protein (3 μM) was immobilized on the streptavidin (SA) sensor and at least three to four concentrations of each affibody were injected to estimate the K_D_ values.

The binding affinities of the affibodies for TMEM16F protein were directly measured using BLI. Since the SBP tag used for affinity chromatography can bind to streptavidin (SA)-conjugated biosensors, hTMEM16F proteins were directly immobilized on the SA biosensor (Figure 4A). After immobilization, various concentrations of affibodies were injected to monitor their association with and dissociation from TMEM16F (Figure 4B). In agreement with the ELISA data, #119 and #50 affibodies showed high binding affinity for TMEM16F at 0.13 ± 0.08 and 0.27 ± 0.04 μM, respectively. #39 and #128 affibodies showed modest binding affinity (0.68 ± 0.04 and 0.96 ± 0.04 μM, respectively), while #140 and #165 showed low binding affinity (12.44 ± 0.41 and 4.26 ± 0.02 μM, respectively). Unlike the results obtained using ELISA, #128 exhibited high affinity 0.96 ± 0.04 μM) in the BLI experiment. This discrepancy may indicate under- or overestimation of the affinities by ELISA. Because anti-affibody antibodies were no longer commercially available, we used mouse anti-His antibody and mouse secondary antibody to detect the binding of candidate affibodies to hTMEM16F. Thus, the affinities estimated using ELISA could be affected by how well the His-tag of each affibody was exposed and this could affect the binding affinities. Next, we examined the specificity of two affibodies (#50 and #119) which have high affinity for the TMEM16F by investigating the binding capacity of them onto the closely related TMEM16 family member, TMEM16A. After purifying the human TMEM16A (hTMEM16A) protein which tagged with SBP tag, hTMEM16A proteins were directly immobilized on the SA biosensor. Then, various concentrations of affibodies were injected. Unlike TMEM16F, it was confirmed that these affibodies could not bind to TMEM16A well (Supplementary Figure S3). Taken together, the results of the various independent experiments show that the candidate affibodies bound to TMEM16F.

### 2.5 Validation of candidate affibodies binding to the hTMEM16F in lipid-environment

**FIGURE 5:**
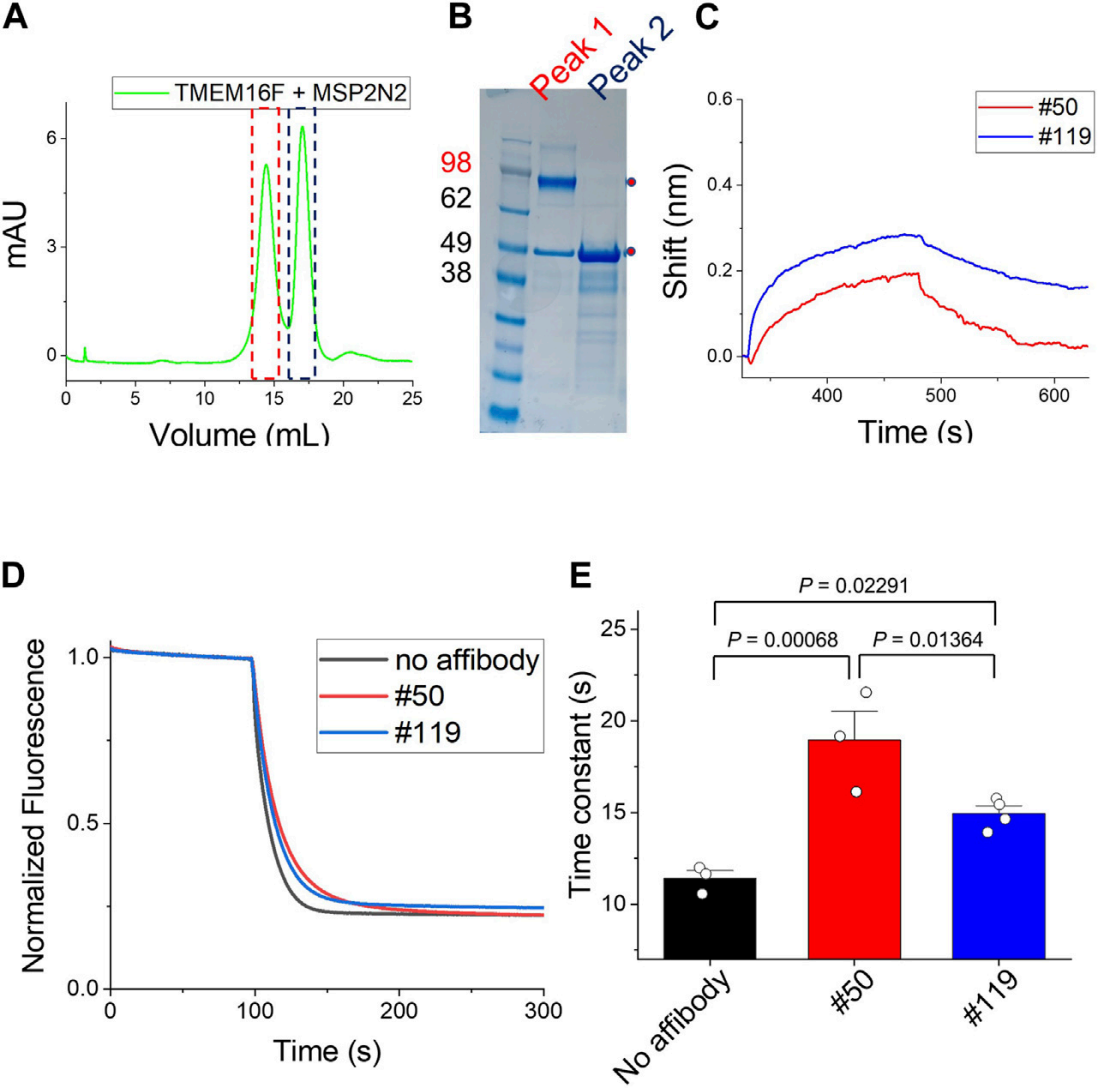
Binding of affibodies to TMEM16F in a lipid environment **(A)** and **(B)** Separation of the nanodisc complex containing TMEM16F from the empty nanodisc by FPLC under detergent-free conditions. The two peaks in the FPLC profile were confirmed by Coomassie blue staining of SDS-PAGE gels. **(C)** The association and dissociation profiles of #119 and #50 affibodies (10 μM) from the TMEM16F nanodisc immobilized on streptavidin (SA)-conjugated biosensor chips were investigated. **(D)** Functional effects of #50 and #119 affibodies on the TMEM16F in the liposomes. 20 μM of each affibody was applied in the assay. Scrambling activity was measured in the presence of 0.5 mM Ca^2+^. **(E)** The time constant of fluorescence decay from the scrambling assay was calculated by fitting the traces with single exponential function. Data are presented as mean ± SEM. One-way ANOVA analysis was performed and *p*-values between control (no affibody), #50 and #119 affibody treatment were calculated by Fisher’s test using Origin 2020 (OriginLab).

Detergent is mandatory for the purification of TMEM16F. In the BLI experiments, we immobilized TMEM16F protein in detergent micelles on streptavidin (SA)-immobilized sensors and then injected various concentrations of candidate affibodies. To minimize the effect of detergent in the BLI, we incorporated TMEM16F protein into a nanodisc using the membrane scaffold protein (MSP), 2N2 (Bayburt et al., 2002; Bayburt and Sligar, 2010). By adjusting the ratios of TMEM16F, MSP, and lipid, we were able to separate the nanodisc complex containing TMEM16F from the empty nanodisc (Figures 5A, B). After immobilizing 3 μM TMEM16F-nanodisc complex on the streptavidin-conjugated (SA) biosensors, 10 μM #119 and #50 affibodies, previously shown to have high binding affinities in ELISA, were injected to monitor their association and dissociation constants. Finally, we confirmed that two affibodies, #119 and #50, could bind to TMEM16F in a lipid environment (Figure 5C).

Further, we investigated the effects of affibodies on the scrambling activity by using proteoliposome. 20 μM of each affibody was applied to both side of the liposomes during the scrambling assays. In the presence of affibodies, the scrambling activity was slightly reduced (Figure 5D). No dramatic changes were observed in the fluorescence signal reduction, but the speed of lipid transport slowed down. When we calculated the time constant of fluorescence decay in each condition by fitting the traces with single exponential function, the treatment of affibody affected the scrambling with slightly different kinetics, τ (no affibody, 11.4 ± 0.4), τ (#50, 18.9 ± 1.6) and τ (#119, 14.9 ± 0.4) (Figure 5E).

### 2.6 Detection of TMEM16F in cells by using candidate affibodies

**FIGURE 6:**
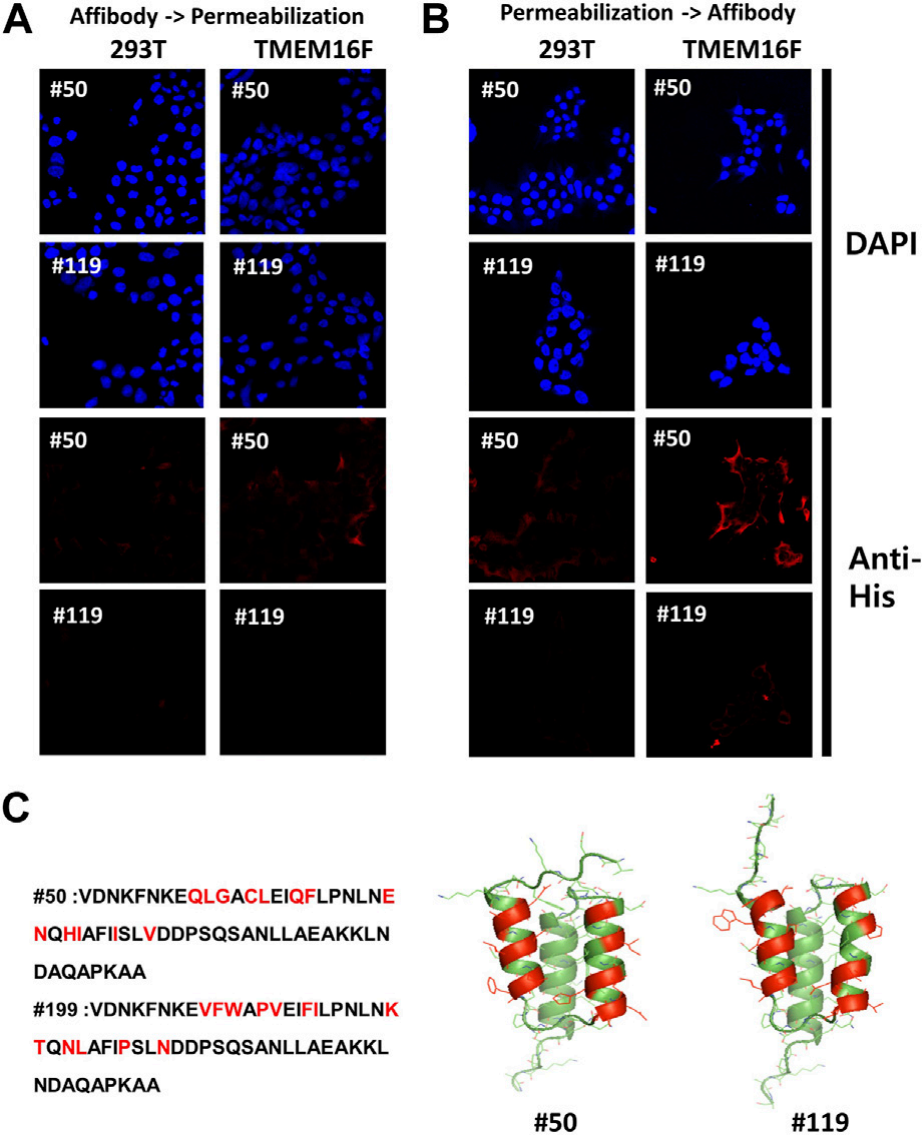
Binding capability of candidate affibodies to the TMEM16F in the cells **(A)** and **(B)** Immunofluorescence staining of 293T cells by using candidate affibodies. Non-transfected and TMEM16F expressing 293T cells were imaged. Affibodies were applicated to the cells before **(A)** and after **(B)** the membrane permeabilization. For the detection of affibodies, Alexa 555 conjugated anti-His antibody was treated. **(C)** Predicted structures of candidate affibodies. Structural prediction of affibody was performed using AlphaFold. The region could bind to the TMEM16F was indicated with red color in both sequences and the structures.

Finally, we tried to detect the endogenous and exogenous TMEM16F in 293T cells by using candidate affibodies. To do this, immunofluorescence staining and confocal imaging were performed. When considering the future application, it is very important to know where the binding site of affibody on the TMEM16F is. To test whether the binding site of affibodies exists in the extracellular or intracellular side of TMEM16F, we applicated the affibodies to the cells before and after the membrane permeabilization step. #50 affibody could stain both endogenous TMEM16F in 293T cells and overexpressed TMEM16F without permeabilization (Figure 6A). More stained signal was observed in the TMEM16F-overexpressed cells. However, we could not detect any signals from the #119 affibody staining. Next, we applied the affibodies after the membrane permeabilization. For #50 affibody, similar results were monitored from 293T cells and TMEM16F overexpressed cells (Figure 6B). Interestingly, we could detect fluorescent signal from the TMEM16F overexpressed cell using #119 affibody after membrane permeabilization. From these results, we could notice that the binding site of #50 and #119 exist in the extracellular- and intracellular side of TMEM16F, respectively.

## 3 Discussion

Although TMEM16F scramblase is involved in important physiological processes, only limited research has been done to identify modulators of TMEM16F function. To obtain new molecules that target TMEM16F, we conducted phage display screening for affibodies. Since we used the purified whole TMEM16F protein, most of the affibody candidates were expected to bind to the TMEM16F protein in ELISA and BLI. By comparing the binding of candidate antibodies to hTMEM16F-coated and streptavidin only-coated plates, 98 clones were selected for sequencing, which yielded 46 perfect sequences. Unfortunately, among the 10 initial candidates, three candidates were not expressed despite more than three attempts. We think that these candidates might be stable when part of the bacteriophage but not when they are expressed in *E. coli*. Interestingly, the predicted structures of affibodies were almost similar to the wild-type or parental affibody (Figure 6C). Thus, we think our affibodies also share the merits of general affibodies such as high stability and easy penetration.

Affibodies could have potential in therapeutical and diagnostic applications (Lofblom et al., 2010). Considering that TMEM16F is involved in cellular processes, TMEM16F-specific affibodies can be used for the treatment of many diseases. Although TMEM16F is not the only protein which transports PS, PS is highly exposed on the plasma membrane of cancer cells (De et al., 2018). High PS in cancer cells can be triggered by the inhibition of flippases and the activation of scramblases. Unlike other scramblases, PLSCRs and TMEM16 proteins exhibit Ca^2+^-induced non-apoptotic PS exposure on various cancer cells (Behuria et al., 2022). Exposed PS on tumor cells and tumor cell-derived microvesicles could drive the immunosuppressive effect of tumor cells on immune cells and increase the growth and metastasis of tumor cells (Birge et al., 2016). Thus, modulation of PS distribution could represent a new target for cancer treatment (Chang et al., 2020; Behuria et al., 2022). TMEM16F is the most abundant TMEM16 family member in microglia cells, and its function is important for the phagocytic activity of microglia (Batti et al., 2016). Since modulation of microglia function is a new target for the treatment of neurodegenerative diseases caused by malfunction or hyperactivity of microglial cells, TMEM16F could be potentially targeted to regulate the function of microglia. Finally, TMEM16F has been proposed as a new target for the inhibition of SARS-CoV-2 virus cell entry and syncytia formation induced by SARS-Cov-2 infection (Braga et al., 2021). PS exposure by TMEM16F is critical for virus infection and its replication (Zaitseva et al., 2017). Thus, finding new modulators of TMEM16F function could lead to new therapies for virus infections, including HIV, Ebola, and Cov-2 virus.

Through the functional assay using proteoliposomes, we were able to confirm that screened affibodies in this study could affect the function of TMEM16F modestly. Despite of small impact, since the affibodies have TMEM16F-specificity which surpasses other protein, such as TMEM16A (Supplementary Figure S3), they may still be valuable for therapeutic purposes. In case of #50 affibody, its binding site seems to exist in the extracellular side of TMEM16F. This fact provides the advantage for the delivery of the affibody to the cell when considering therapeutical applications. Especially, this affibody can be used as a targeting ligand that can delivery therapeutic drugs such as radionuclides, toxins, and nanoparticles (Stahl et al., 2017). Therefore, the modest inhibition observed might be enhanced when the affibodies are used in combination with other drugs or treatments.

Since the affibodies tested in this study were not the optimized affibodies, the binding affinity and inhibition could be increased by conducting maturation processes in the future study (Grindel et al., 2022). Further, if we have the complex structure of TMEM16F-affibody, the affibodies could be engineered to enhance the binding affinity based on the findings on key residues for the affibody binding to TMEM16F.

In summary, we screened novel TMEM16F-specific affibodies via phage display using purified TMEM16F and identified affibodies that bind specifically to TMEM16F with high affinity. Further work will be required to characterize and optimize the affibodies for the regulation of TMEM16F function so that they can be used for therapeutical purposes.

## 4 Materials and methods

### 4.1 Purification of human TMEM16F protein

A cDNA clone for Human TMEM16F variant 1 (Accession number, NM_001025356.2) was purchased from Genescript. TMEM16F protein was tagged with SBP at its C-terminus to facilitate its purification. After transfection into GnTI^−^ cells using polyethylenimine (PEI) for 48–72 h, cells were harvested and resuspended in lysis buffer (150 NaCl, 20 HEPES, pH 7.4). The cells were then homogenized with a dounce homogenizer and sonicated for 1 min, and 1% digitonin was added to facilitate extraction of human TMEM16. The extract was bound on a Streptavidin Plus Ultralink column in the presence of 0.06% GDN instead of 1% digitonin, and TMEM16F protein was eluted with 8 mM biotin. TMEM16 protein was further purified by FPLC on a Superose 6 column. The cell lines present in this study were obtained from ATCC (American Type Culture Collection).

### 4.2 Scrambling assay

Liposomes were prepared from a 3:1 mixture of POPC and POPG, and 0.5 mol% NBD-labeled phosphatidylethanolamine (PE) was added to the lipid mixture. Lipids were dissolved in reconstitution buffer (300 mM KCl, 20 mM HEPES, pH 7.4) in the presence of 35 mM 3-((3-cholamidopropyl) dimethylammonio)-1-propanesulfonate (CHAPS). Then, TMEM16F proteins were added at the desired protein to lipid ratio; typically 5 μg protein/mg of lipid was used. Proteoliposomes were formed by removing detergent with Bio-Beads SM-2 Adsorbent (Bio-Rad, Hercules, CA). For scrambling assays, liposomes were extruded through a 400 nm membrane. Phospholipid scrambling activity was measured as described in a previous study (Lee et al., 2016). Briefly, 20 μL liposomes were added to 1.98 mL of scrambling solution (300 mM KCl, 50 mM HEPES, 0.5 mM Ca(NO_3_)_2_ or 2 mM EGTA, pH 7.4). Fluorescent signals were monitored (excitation, 470 nm; emission, 530 nm) with a spectrofluorimeter (HORIBA Scientific, Edison, NJ). Finally, 40 μL of sodium dithionite (40 mM final concentration) was added to bleach the NBD fluorophores. In order to calculate the time constant of fluorescence decay, the traces were fitted with single exponential function. For the statistical analysis, One-way ANOVA was performed by Fisher’s test using Origin 2020 (OriginLab). Data are presented as mean ± SEM.

### 4.3 Biopanning for TMEM16F-specific affibodies

The affibody library was created using a SPA-Z scaffold template and random primers encoding helices 1 and 2 of the Z-domain. The gene fragments were then double-digested with SfiI/NotI and cloned into the pIGT2 phagemid vector provided by IgTherapy Co. The resulting constructs were transformed into *E. coli* ER cells, and the resulting library consisted of 3 × 10^8^ independent affibody clones. To construct the affibody recombinant phage library, M13K07 helper phages (New England Biolabs) were used. The library was screened against purified human TMEM16F protein immobilized on streptavidin-coated 96-well plates. The plates were blocked with PBS containing 2% BSA, and the affibody recombinant phage (1 × 10^11^ plaque-forming units [PFU]) was added and incubated for 1 h at 30 °C. After washing the unbound phages with PBS containing 0.05% Tween20, the bound phages were eluted using 0.2 M glycine-HCl (pH 2.2) and immediately neutralized with 1 M Tris-HCl (pH 9.0). For the next biopanning round, the eluted phages were used to infect *E. coli* ER for 30 min at 37 °C, and then helper phages (1.84 × 10^9^ PFU) were added and incubated for 1 h at 37 °C. The superinfected *E. coli* were cultured in SB medium containing 50 μg/mL ampicillin and 10 μg/mL kanamycin overnight. The input and output phage PFU in all rounds of biopanning were measured. Following the seventh round of biopanning, phage ELISA was conducted. In total, 192 individual colonies were randomly selected, and recombinant phages were prepared. hTMEM16F with streptavidin (target sample) and streptavidin alone (background control) were immobilized on 96-well plates, and individual recombinant phages were then added and incubated for 1 h at 30 °C. After washing the plates three times with PBS containing 0.05% Tween20, HRP-conjugated anti-M13 antibody (Sino Biological) was added and the recombinant phages were identified using TMB (Thermo) as a substrate. Absorbance was measured at 450 nm. Positive clones were identified and subjected to DNA sequencing using a phagemid primer (5′-GAT​TAC​GCC​AAG​CTT​TGG​AGC-3’; Bioneer).

### 4.4 Purification of TMEM16F affibodies

DNAs for 10 candidate affibodies selected by biopanning were synthesized and then incorporated into the bacterial expression vector, pBT7-C-His (Bioneer). Plasmid DNAs were transformed into BL21 (DE3). The cells were grown to an OD of ∼0.6–0.8 and protein expression was induced by the addition of 1 mM IPTG for 3 h. Then, cells were resuspended in lysis buffer (150 NaCl, 20 Tris-Cl, pH 8.0). Proteins were purified using affinity chromatography (Talon resin, Takara). To reduce the non-specific binding, resins were washed with 40 mM imidazole and affibody was eluted with 200 mM imidazole. After removing the remaining imidazole by dialysis overnight, affibody proteins were concentrated using centrifugal filters (Thermo).

### 4.5 Enzyme-linked immunosorbent assay

96-well plates were coated with 50 μL of TMEM16F protein (5 μg/mL) per well at 4°C overnight. After blocking with 2% BSA for 2 h, each well was washed three times with 200 μL of PBST (0.1% Tween 20). Then, 100 μL of affibodies ranging in concentration from 0 to 200 μM was added and incubated for 1 h. To detect His-tagged affibodies, mouse anti-His antibody (1:1000) and alkaline phosphatase-conjugated anti-mouse IgG antibody (1:5000) were used. After each step, the plate was washed three times with PBST. p-nitrophenyl phosphate (PNPP) was used as a colorimetric soluble substrate for ELISA. The absorbance at 405 nm was measured using a microplate reader (Molecular Device).

### 4.6 Bio-layer interferometry (BLI)

For the measurement of binding affinity, K_D_ values for each affibody, Bio-Layer Interferometry BLItz (forteBIO) was used. The Streptavidin (SA) Biosensor was hydrated with deionized water for 10 min. The initial baseline was acquired by incubating the biosensor with buffer containing 150 NaCl and 20 HEPES, pH 7.4, for 30 s. Then 4 μL of TMEM16F (3 μM) tagged with SBP was loaded onto the Octet Streptavidin (SA) biosensors for 150 s. After loading, the biosensor was transferred back to the buffer used for baselining for 150 s. Then, to measure the association of the affibody with TMEM16F protein, 4 μL affibodies were loaded for 150 s. Finally, the dissociation of the complexes was monitored by transferring the biosensor back into buffer containing 150 NaCl and 20 Tris-Cl, pH 8.0. In order to calculate K_D_ value for each affibody, we analyzed the data with the BLItz Pro software (forteBIO) using global fitting (1:1) according to the manufacturer’s instructions. For the global fitting, association and dissociation curves were fitted with following equations.

i) association phase:

$$y=R\max\frac{1}{1+\frac{Kd}{Ka*\left[Analyte\right]}}\left(1-e^{-\left(Ka*\left[Analyte\right]+Kd\right)x}\right)\text{ and }R_{\max}=\text{maximum binding signal}$$

ii) dissociation phase:

$$y=y0e^{-Kd\left(x-x0\right)}\text{ and }y0=R\max\frac{1}{1+\frac{Kd}{Ka*\left[Analyte\right]}}\left(1-e^{-\left(Ka*\left[Analyte\right]+Kd\right)x0}\right)$$

Finally, K_D_ values were derived from the equation, $D=\frac{\left[A\right]\left[B\right]}{\left[AB\right]}=\frac{Kd}{Ka}$ . K_D_ values are presented as mean ± SEM (n = 3).

### 4.7 Incorporation of hTMEM16F proteins into nanodisc complex

Purified hTMEM16F proteins were incorporated into nanodiscs composed of POPC and POPG mixture at a molar ratio of 3:1. The lipid mixture was solubilized with 10 mM DDM. For the purification of membrane scaffold protein (MSP) 2N2, plasmid DNA (addgene, #29520) was transformed into BL21(DE3). After induction of proteins by adding 1 mM IPTG, 2N2 proteins were purified by using Ni-NTA resin (Qiagen). The mixture of hTMEM16F:MSP 2N2:lipid at a molar ratio of 1:5:100 was incubated with Bio-Beads SM-2 Adsorbent to remove the detergent. To separate empty nanodisc and TMEM16F containing nanodisc complex, the mixture was further purified by FPLC on a Superose 6 column.

### 4.8 Immunofluorescence staining

293T cells seeded on poly-L-lysine (PLL)-coated coverslips were transfected with human TMEM16F and fixed with a solution containing 4% (w/v) paraformaldehyde (PFA), 1× PBS, and pH 7.4 for 8 min. The fixed samples were divided into two groups for the application of affibody before and after the permeabilization step. For the permeabilized conditions, cells were incubated with permeabilization solution (0.2% (v/v) Triton X-100 and 1× PBS, pH 7.4) for 10 min and then with blocking solution (3% (w/v) BSA, 0.1% (v/v) Tween-20 and 1× PBS, pH 7.4) for 1 h. Candidate affibodies (#50 and #119) prepared in blocking solution (5 ng/μL) were added for 1 h. The cells were then washed three times with washing solution (0.1% (v/v) Tween-20 and 1 × PBS, pH 7.4) and incubated with Alexa-555 conjugated anti-His antibody (12.5 μg/mL). Confocal images were obtained using an inverted Nikon ECLIPSE Ti-E confocal microscope.

### 4.9 *In silico* three-dimensional models and visualization

In silico three-dimensional structure prediction of affibodies (#50 and #119) were performed using the online Colab version of AlphaFold2 (Mirdita et al., 2022). The predicted model structures were visualized using the PyMol Molecular Graphics System (Version 2.4, Schrödinger, NY, United States).

## Data Availability

The original contributions presented in the study are included in the article/Supplementary Material, further inquiries can be directed to the corresponding authors.
