# Supplementary material for: Generation of human TMEM16F-specific affibodies using purified TMEM16F
Source: Front Mol Biosci. 2024 Jan 11;10:1319251. doi: 10.3389/fmolb.2023.1319251 (PMC10808743; doi:10.3389/fmolb.2023.1319251)
Supplement: Supplementary file 1 [file Table1.DOCX]

Supplementary Material


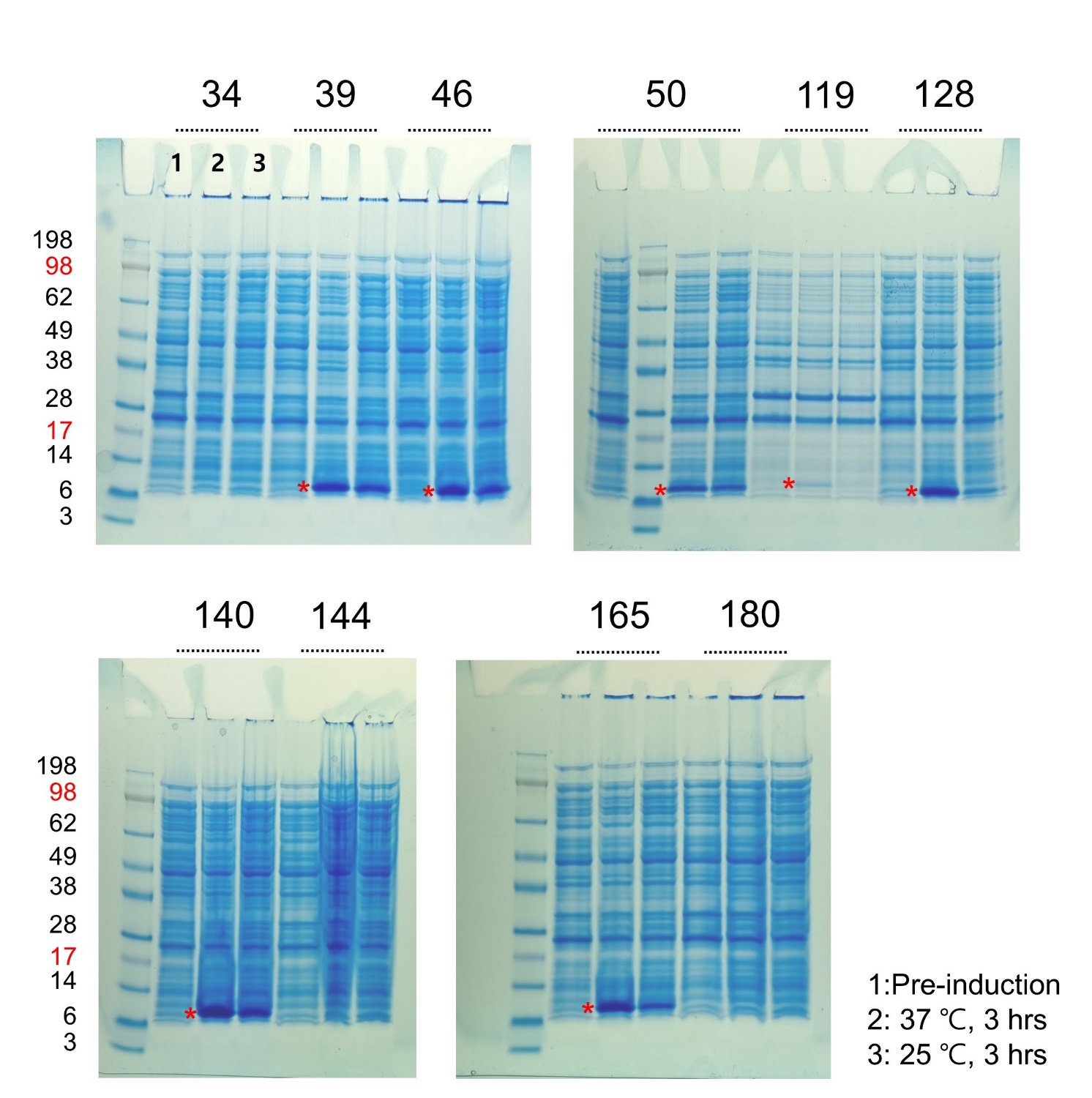


**Supplementary Figure 1.** **Validation of the expression of the candidate affibodies expressed in small scale cultures.** Test used to validate the expression of the candidate affibodies. Proteins were induced by IPTG at 37°C and 25°C for 3 hours.


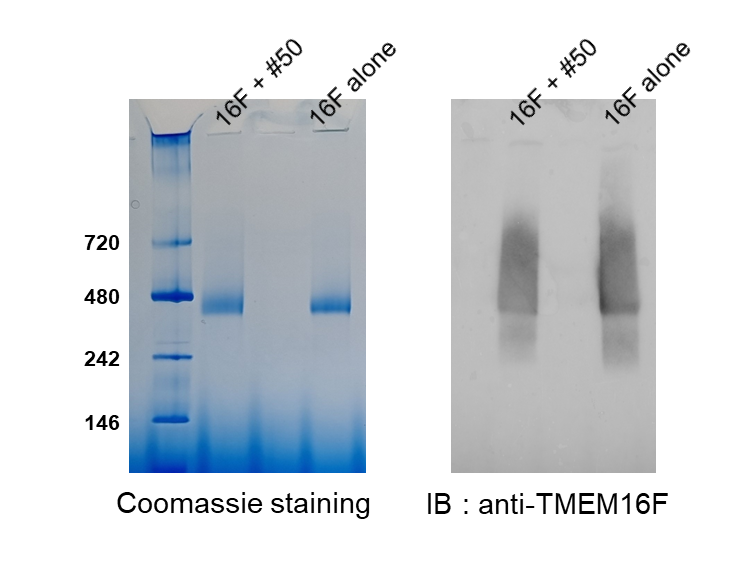


**Supplementary Figure 2.** **Blue Native PAGE followed by western blot analysis.** The Affibody and TMEM16F were mixed at 4℃ for 3 h, and then separated by Superose 6 FPLC. After FPLC, the presence of TMEM16F was confirmed by Coomassie blue staining of Blue Native PAGE gels and western blotting.

TMEM16F

TMEM16A

TMEM16F

TMEM16A

**Supplementary Figure 3.** **Specificity of candidate affibodies.** To test the specificity of #50 and #119 affibodies on TMEM16F, closely related TMEM16 family member, TMEM16A protein was expressed and purified. After immobilizing the TMEM16A protein into the BLI sensor, binding of candidate affibodies to the TMEM16A was monitored.
